# Understanding Health Literacy Through Patients' Interpretation of Health Education Leaflets: A Thematic Narrative Review

**DOI:** 10.1111/hex.70479

**Published:** 2025-11-04

**Authors:** Yung‐Hui Tang, Chin Tsai Lin, Li‐Chu Wu

**Affiliations:** ^1^ Department of Nursing Kaohsiung Veterans General Hospital Kaohsiung Taiwan; ^2^ Ph.D. program of Curriculum and Teaching, Department of Education National University of Tainan Tainan Taiwan; ^3^ Department of Education National University of Tainan Tainan Taiwan

**Keywords:** cognitive learning theories, health communication, health literacy, patient education materials, patient‐centred care, user‐centred design

## Abstract

**Background:**

Understanding how patients interpret health education leaflets is essential in promoting equitable and effective health communication. This narrative thematic review synthesises current evidence and introduces a conceptual model to inform patient‐centred leaflet design.

**Methods:**

Twenty‐eight English‐ and Chinese‐language studies (2010–2024) were identified via PubMed, CINAHL, ERIC, Airiti Library and Google Scholar. Boolean searches combined terms related to health literacy, patient education materials and information design. Eligible studies underwent open coding and thematic synthesis, guided by Nutbeam's three‐tier health literacy model (functional, interactive and critical) and cognitive learning theories (Cognitive Load, Multimedia Learning and Dual Coding).

**Results:**

Patients frequently encountered dense text, medical jargon and poor text–image integration. Thematic mapping aligned barriers with literacy levels, generating two strategy tables. The proposed ‘Boiling Water Model’ depicts the progression from information gathering to value integration and a ‘boiling point’ of insight, supported by reflective prompts, risk–benefit comparisons and clarifying aids.

**Conclusions:**

Embedding cognitive learning principles within leaflet design can facilitate patients' progression towards critical health literacy. A theory‐driven, user‐centred approach—incorporating plain language, interactive cues and reflective elements—can transform leaflets into dynamic tools that enhance comprehension and foster informed, value‐aligned health decisions.

**Patient or Public Contribution:**

No patients or members of the public were directly involved in the design, conduct or analysis of this narrative review. The study focus and synthesis were guided by documented patient experiences and reported barriers to comprehension in the existing literature, informing patient‐centred health education material design.

**Clinical Trial Registration:** Not applicable. This study is a narrative literature review and does not report results of a clinical trial.

**Abbreviations**
CLT: Cognitive Load Theory
DCT: Dual Coding Theory
HEX: *Health Expectations*
MMLT: Multimedia Learning Theory
PEC: *Patient Education and Counselling*
PILs: Patient Information Leaflets
PRISMA: Preferred Reporting Items for Systematic Reviews and Meta‐Analyses

## 1 Introduction

Health literacy is a core competency that enables individuals to make informed health decisions and actively participate in self‐care [1, 2]. In contemporary healthcare systems—where patient autonomy and shared decision‐making are increasingly emphasised—the ability to understand and apply health information has become a key determinant of health outcomes [3].

Patient education leaflets (PILs) are among the most common tools for delivering health information, encompassing topics such as disease management, medication use, postoperative care and preventive measures. However, understanding the textual and visual content of these materials is the foundation upon which patients can practice health literacy [4]. While numerous patients possess basic reading skills, they may struggle to interpret medical terminology, connect visuals with relevant text or recognise critical safety warnings. Such misunderstandings can lead to medication errors, misinterpretation of care instructions or delays in seeking appropriate treatment, ultimately affecting individual health outcomes and the efficiency of healthcare delivery.

Given these challenges, it is crucial to update and redesign PILs based on the latest evidence. Recent evidence underscores why updating PILs remains a priority. Contemporary health literacy frameworks highlight people's ability to locate, understand and use information to make health decisions; however, large segments of the public struggle with written health materials that are often written above recommended reading levels [5, 6]. At the same time, infographic‐based PILs have been found to improve comprehension, navigability and user satisfaction more than traditional text formats [7]. Beyond grade level, recent guidance indicates the importance of layout, imagery, colour and content hierarchy to reduce cognitive load and leverage dual‐channel processing during reading [8]. Evidence on accessibility and multilingual availability similarly suggests that patient‐facing materials often lack these qualities [6], reinforcing the need for design‐guided improvements. These developments strengthen the rationale for a theory‐informed approach to PIL design, grounded in Cognitive Load Theory [9], Multimedia Learning Theory [10] and Dual Coding Theory [11]. This motivates our thematic narrative synthesis that focuses on how patients read and interpret health education leaflets (see also Don Nutbeam's three‐level model of health literacy) [1].

This review adopted a thematic narrative approach to synthesise and analyse 28 Chinese‐ and English‐language studies published between 2010 and 2024. The analysis focuses on patients' cognitive processes while reading leaflets, relevant reading theories, and the application of Nutbeam's three‐tier health literacy model—functional, interactive and critical literacy—to patient education. This model spans basic comprehension skills, to applying information in communication, to critically evaluating health information for autonomous decision‐making [1].

To further explain barriers in comprehension, the review integrates cognitive learning frameworks, including Cognitive Load Theory [9], Multimedia Learning Theory [10] and Dual Coding Theory [11]. These theories highlight how excessive cognitive demands, poorly structured information or misaligned visuals and text can impede understanding, reduce recall and reduce the effectiveness of educational materials.

By combining insights from health literacy research and cognitive psychology, this review identifies practical design strategies—including simplifying language, structuring visual layouts, prioritising messages and fostering reader engagement—that can make leaflets more accessible and meaningful. The findings can guide healthcare professionals, educators and public health institutions in generating patient education tools that are interactive, adaptable and sensitive to diverse literacy levels, promoting informed decision‐making and positive health behaviours.

## 2 Methods

### 2.1 Search Strategy and Selection Criteria

This study adopted a thematic narrative review approach, rather than a full systematic review, to explore how patients interpret health education leaflets in relation to health literacy. While the search and selection process was reported with reference to PRISMA guidelines [12, 13] to enhance transparency, the aim was to synthesise thematic insights rather than to perform a systematic synthesis or meta‐analysis.

We conducted a systematic search of five databases—PubMed, CINAHL, ERIC, Airiti Library and Google Scholar—for studies published between January 2010 and April 2024. Boolean search strategies combined terms related to health literacy, patient education materials and reading comprehension. The complete search strings were as follows:

PubMed/CINAHL/ERIC:

(“health literacy”[Title/Abstract] OR “patient literacy”[Title/Abstract]) AND (“patient education materials”[Title/Abstract] OR “leaflet”[Title/Abstract] OR “brochure”[Title/Abstract]) AND (“reading”[Title/Abstract] OR “comprehension”[Title/Abstract] OR “visual design”[Title/Abstract])

Airiti Library/Google Scholar (Chinese):

(健康識能 OR 健康素養) AND (病人衛教單張 OR 病人衛教手冊) AND (閱讀 OR 理解 OR 視覺設計).

### 2.2 Screening and Selection Process

Inclusion criteria were: (1) peer‐reviewed articles in English or Chinese; (2) studies focusing on health literacy, reading comprehension or the design of patient education materials; and (3) empirical research, tool development, theoretical analyses or design principle studies.

Exclusion criteria were: (1) psychometric validation studies only without content analysis; (2) conference abstracts or non‐peer‐reviewed sources; and (3) inaccessible full texts.

A total of 982 records were retrieved. After removing 312 duplicates, 670 titles and abstracts were screened, of which 489 were excluded for irrelevance to health literacy or patient education materials. The remaining 181 full‐text articles were assessed, and 153 were excluded due to psychometric‐only focus without content analysis (*n* = 72), conference abstracts or non‐peer‐reviewed sources (*n* = 54), or inaccessible full texts (*n* = 27). Finally, 28 articles met all inclusion criteria.

Two independent reviewers (Author A and Author B) conducted the screening process. Disagreements were resolved through discussion, and a third reviewer (Author C) adjudicated unresolved conflicts. Inter‐rater agreement during full‐text screening reached 0.86 (Cohen's *κ*) [14].

**Figure 1:**
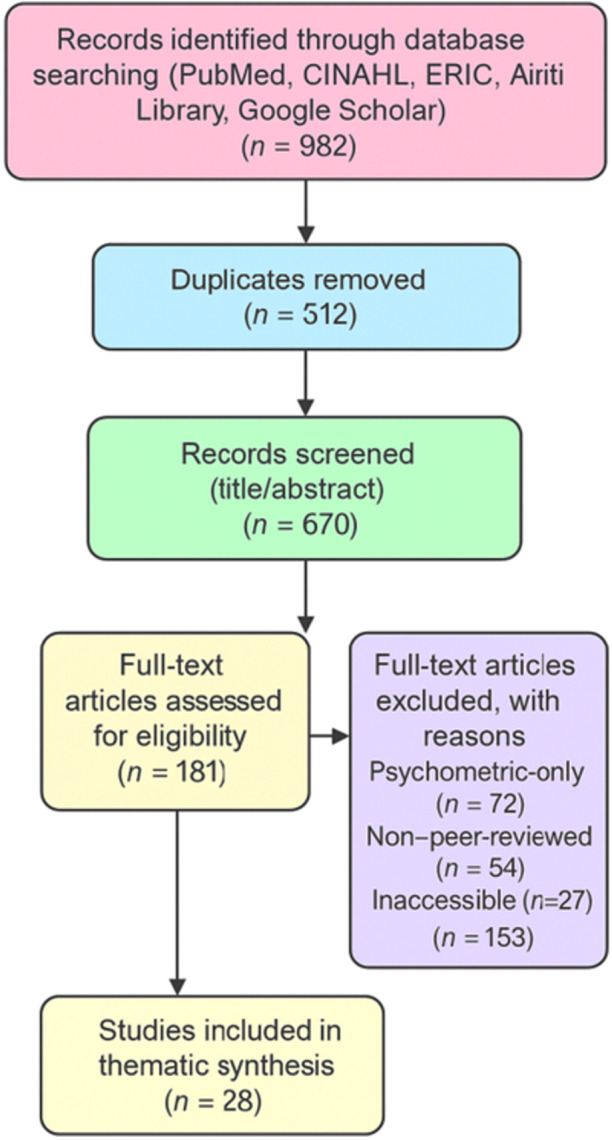
PRISMA flow diagram of the study selection process.

The study selection process is illustrated in Figure 1, following the PRISMA flow diagram. The diagram was generated based on the R code kindly provided by the reviewer, using the final numbers of records identified, screened, excluded (with reasons) and included in this thematic narrative review. A summary of the 28 studies included in the thematic synthesis is provided in Appendix.

From the 982 records identified through database searching, 312 duplicates were removed. A total of 670 titles and abstracts were screened, 489 were excluded, and 181 full‐text articles were assessed for eligibility. Of these, 153 were excluded (72 psychometric only, 54 non‐peer‐reviewed and 27 inaccessible full text), leaving 28 studies included in the thematic synthesis.

### 2.3 Thematic Analysis

Using open coding [15], thematic dimensions were synthesised across the included studies, focusing on: (1) applications and challenges of: contentReference[oaicite:2]{index=2}'s three‐level health literacy model; (2) comprehension barriers in reading PILs; and (3) implications of cognitive learning theories—:contentReference[oaicite:3]{index=3}, :contentReference[oaicite:4]{index=4}, and:contentReference[oaicite:5]{index=5}—for educational material design.

## 3 Results

To synthesise practical implications from the included studies, we first mapped how selected cognitive learning theories can inform the design of PILs (Table 1). These theories explain how patients process information and highlight strategies to optimise comprehension and reduce cognitive overload.

**Table 1:** Cognitive learning theories and design implications for patient education leaflets.

Learning theory	Core concept	Design implications
Cognitive load theory	Working memory has limited capacity; instructional design should minimise unnecessary cognitive burden.	Simplify sentence structure, reduce visual or textual distractions, and segment and sequence information logically.
Multimedia learning theory	Coordinated text and visuals enhance meaning‐making and prevent the split‐attention effect.	Align text and images, use concrete and relevant visuals, and provide labels and guiding cues.
Dual coding theory	Text and visuals are processed through complementary cognitive channels, improving memory retention.	Combine diagrams with keywords, highlight key points and avoid text‐only presentations.

*Note:* Selected cognitive learning theories can guide leaflet design by optimising comprehension, integrating verbal and visual information, and reducing cognitive overload.

We aligned these theoretical principles with Don Nutbeam's (2000) three‐level health literacy model to develop a set of design strategies tailored to different literacy levels (Table 2). Tables 1 and 2 provide an overview framework linking learning theories with concrete design approaches, which guided the subsequent thematic synthesis.

**Table 2:** Health literacy levels and recommended leaflet design strategies.

Health literacy level	Definition	Recommended design strategies
Functional literacy	Ability to understand basic health information (e.g., medications and procedures).	Use plain language, provide step‐by‐step instructions and apply clear, readable typography.
Interactive literacy	Ability to communicate with healthcare providers and clarify health needs.	Include question prompts, blank spaces for notes and ‘ask your doctor’ reminders.
Critical literacy	Ability to evaluate information and make autonomous decisions.	Present optional information, explain risks and benefits, and emphasise the ‘right to choose’.

*Note:* Each level of health literacy is linked to practical design strategies for developing patient education leaflets, aiming to enhance accessibility, clarity and patient‐centred communication.

### 3.1 Multilevel Structure of Health Literacy

Health literacy extends beyond the ability to read and understand health information; it encompasses the capacity to engage in health‐related decision‐making and take appropriate action. Nutbeam's three‐level model [1] classifies health literacy as follows:

Functional literacy: basic reading and writing skills that enable individuals to understand healthcare information such as medication instructions, examination procedures and clinic visit guidelines.

Interactive literacy: advanced cognitive and social skills that support active clarification of information, communication of health needs and adaptation to different contexts.

Critical literacy: the ability to critically analyse, evaluate and integrate health information from multiple sources to make informed, value‐aligned decisions.

PILs primarily target functional literacy, offering basic, unidirectional information. Materials designed to foster interactive or critical literacy remain scarce, limiting patients' ability to apply and evaluate information effectively. Designing tools that address all three levels is therefore a pressing challenge.

### 3.2 Challenges in Reading Health Education Leaflets

Leaflets are widely used in clinical and public health settings for education, promotion and prevention due to their low cost and broad reach. However, patients, even those with basic reading skills, often encounter significant comprehension barriers, which can reduce educational effectiveness and compromise the quality of care.

Three main domains emerged from the synthesis as follows:

Linguistic and terminology barriers: use of medical jargon (e.g., ‘PRN’ and ‘NPO’) and complex grammar can confuse patients [16]. Abstract icons or poorly integrated visuals may further hinder understanding unless paired with clear textual explanations [17].

Influence of patient background factors: age, education, language proficiency, cognitive function and culture all influence reading ability [18]. Older adults or those with low literacy often struggle with small fonts, dense text or unclear headings [19]. Cultural and linguistic mismatches can also impair comprehension.

Visual design and text–image integration: layout, colour and image quality directly affect usability. Cluttered designs, abstract visuals and poor sequencing can cause overload [4]. Stress or illness can further limit attention, making a clear visual hierarchy essential.

### 3.3 Implications of Reading Theories for Leaflet Design

Reading a leaflet is a multimodal, cognitively complex process that involves text, images and symbols. This review applies three cognitive learning theories to derive design implications:

Cognitive Load Theory [20]: manage intrinsic, extraneous and germane load by simplifying language, aligning visuals with text and using highlights or worked examples.

Multimedia Learning Theory [21]: integrate verbal and visual information to avoid split‐attention, align illustrations with text, use pointer lines and maintain consistent visual cues.

Dual Coding Theory [11]: pair text with relevant imagery to create two memory pathways and use contextual visuals to anchor meaning and support retention.

### 3.4 Synthesis and Future Research Directions

**Figure 2:**
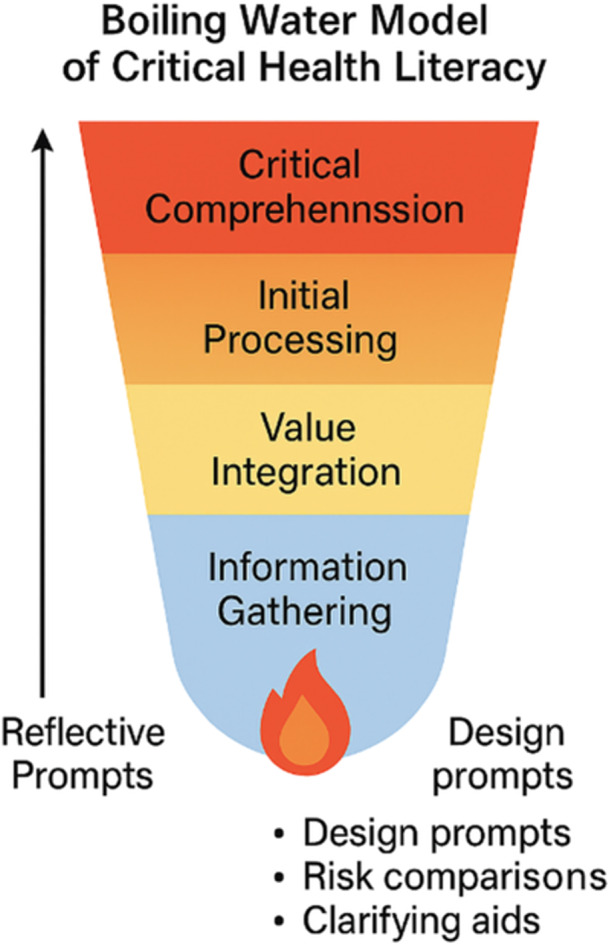
Boiling water model of health literacy. *Note:* This conceptual model illustrates four cognitive stages in patients' engagement with health information: information gathering, value integration, initial processing and critical comprehension. The final stage represents the ‘boiling point’ of health literacy, where reflective insight and decision‐making converge. Design elements such as reflective prompts, risk–benefit comparisons and clarifying aids act as cognitive ‘heat sources’ that sustain engagement and support decisions aligned with personal values.

Patients' interpretation of health education leaflets is shaped by literacy level, cognitive load and text–image integration. Current materials often prioritise functional literacy, overlooking interactive and critical dimensions. Future designs should align with Nutbeam's model and cognitive learning principles, incorporating the following: Plain language and integrated visuals for functional literacy. Prompts and note spaces for interactive literacy. Decision aids, benefit–risk tables and comparative visuals for critical literacy. This review introduces the ‘Boiling Water Model’ (Figure 2) to illustrate the cognitive progression from information gathering, to value integration and to actionable decisions; the model mirrors the gradual heating and transformation of water. This model emphasises that sustained cognitive support is essential for patients to transition from information reception to empowered action.

### 3.5 Boiling Water Model

This conceptual model outlines a progressive path through which patients engage with health information, moving from basic information collection and meaning‐making, to integrating values and actively processing content, and ultimately reaching a stage of critical comprehension. At this final ‘boiling point’, patients achieve reflective understanding that informs their health choices. Features such as reflective prompts, risk–benefit tables and clarifying aids serve as cognitive drivers that maintain attention and foster alignment between decisions and personal values.

Design elements such as reflective prompts, risk–benefit comparisons and clarifying aids act as cognitive ‘heat sources’ that sustain engagement and support decisions that align with personal values. The boiling water model may be particularly relevant in multilingual or multicultural contexts, where comprehension barriers are compounded by linguistic diversity. For instance, adaptations might integrate community health workers to facilitate discussion‐based use of leaflets, as in UK NHS cancer screening initiatives or Australia's culturally tailored diabetes programmes. These cross‐context adaptations demonstrate the model's potential to bridge cultural gaps and enhance patient agency.

When PILs focus solely on transmitting information through simplified instructions, they may fail to sustain the cognitive processes required for deeper integration and critical reasoning. Embedding reflective prompts—such as treatment option comparisons, risk–benefit reflections and value clarification questions (e.g., ‘Does this treatment align with my lifestyle?’)—can stimulate active engagement and promote the development of critical health literacy [22].

## 4 Discussion

This review synthesises how patients interpret health education leaflets, revealing three main thematic dimensions: (1) application and challenges of Don Nutbeam's three‐level health literacy model [1], (2) comprehension barriers in reading leaflets and (3) implications of cognitive learning theories—Cognitive Load Theory [2], Multimedia Learning Theory [3] and Dual Coding Theory [4]—for leaflet design. Building on the findings, this discussion outlines the broader significance of patient‐centred design in health communication. It underscores how future interventions can extend beyond basic information delivery to actively promote critical health literacy.

### 4.1 From Information Delivery to Meaningful Understanding

The findings of this review suggest that many health education leaflets still operate primarily as tools for one‐way information transmission, which often results in superficial reading and low engagement. Patients may not integrate the provided information into their personal health decision‐making, leading to limited behavioural changes. This finding highlights the need to shift from a purely functional literacy approach—where patients only absorb facts—towards fostering interactive and critical literacy [1], where patients can evaluate, question and apply information in their real‐life contexts. This shift requires materials that are not only accurate but also accessible, relatable and cognitively engaging [9, 10, 11].

### 4.2 Establishing Evaluation Mechanisms for Leaflets

Despite growing attention to readability, few studies systematically evaluate how patients actually comprehend and use leaflet content. These findings underscore the necessity of integrating evaluation mechanisms—such as comprehension testing, teach‐back methods and usability studies—into the development and revision cycles of patient materials [9, 10, 11]. These approaches can help to ensure that design choices are guided by patient feedback rather than by assumptions from healthcare professionals alone.

### 4.3 Promoting Interdisciplinary Collaboration and Design Thinking

Enhancing PILs will require collaboration among healthcare professionals, health literacy experts, designers and behavioural scientists. This review supports the call for design thinking approaches, where iterative prototyping, co‐design workshops and human‐centred feedback loops are used to tailor content [10, 11, 23]. This collaborative approach can reduce cognitive load, improve visual–verbal integration and strengthen patients' ability to navigate complex health decisions.

### 4.4 Integrating Theories to Support Critical Health Literacy

The review further highlights the value of using theoretical frameworks to guide leaflet design. Cognitive Load Theory emphasises reducing unnecessary cognitive burden by simplifying layouts and chunking information (i.e., grouping related content into meaningful units) [9]. Multimedia Learning Theory underscores the coordinated integration of text and visuals, ensuring that both modes complement rather than compete with each other in supporting comprehension [10]. Dual Coding Theory highlights that information is more effectively retained when it is processed through both verbal and visual channels, creating parallel pathways that strengthen memory [12]. Together, these frameworks provide complementary perspectives that support the progression from functional towards critical health literacy [1, 9, 10, 12].

**Figure 3:**
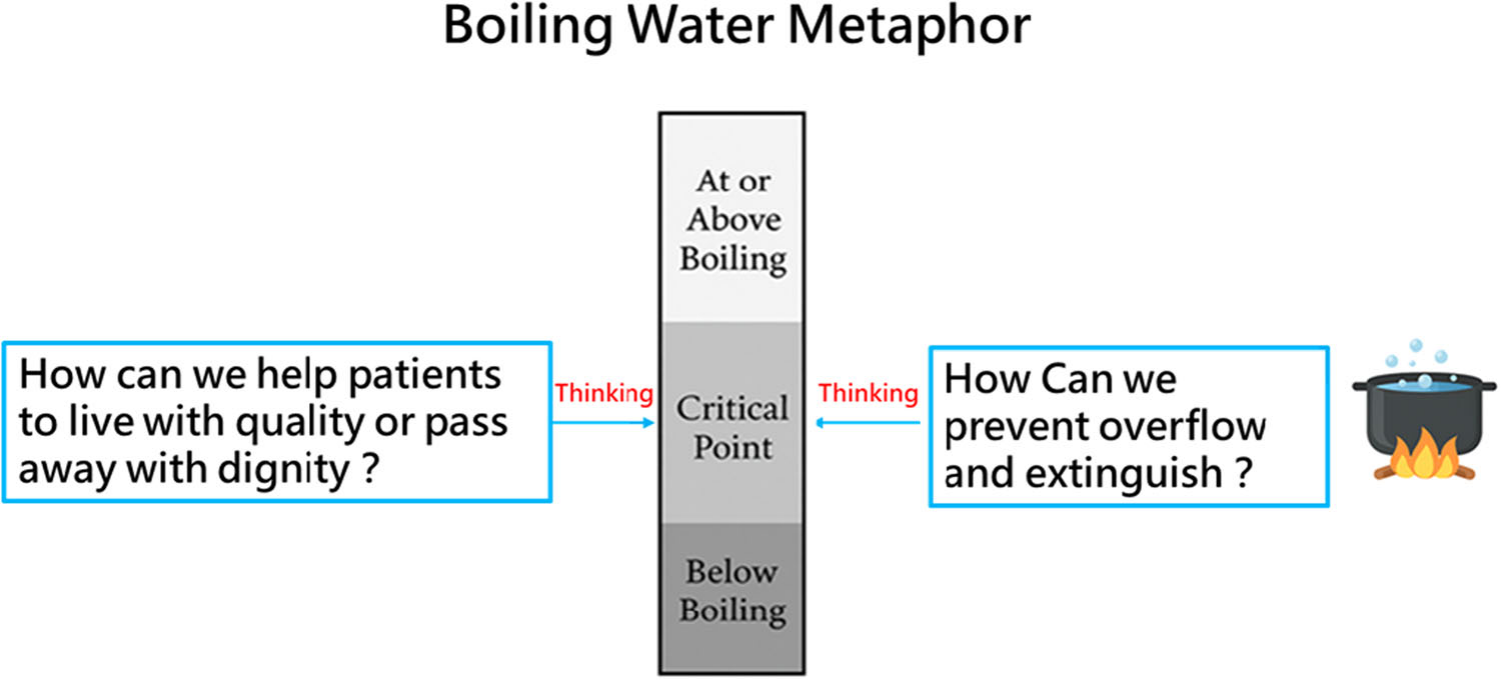
Philosophical extension of the boiling water model: Patient thresholds for quality of life and dignity in dying.

Incorporating reflective prompts—such as treatment option comparisons, risk–benefit reflections and value clarification questions (e.g., ‘Does this treatment align with my lifestyle?’)—can stimulate active engagement and promote the development of critical health literacy. Conceptually, Figure 3 extends the boiling water model into the ethical domain of critical health literacy, illustrating how reflective reasoning enables patients to navigate thresholds of quality of life and dignity in dying. This theoretical expansion serves as a philosophical framework rather than an empirical finding, emphasising how critical health literacy supports patients' autonomous reflection on life quality and end‐of‐life dignity.

### 4.5 Study Limitations

This thematic narrative review has several limitations. First, as a narrative rather than systematic review, it does not employ quantitative meta‐analysis or formal risk‐of‐bias assessment, which may limit reproducibility. Second, only English‐ and Chinese‐language studies published between 2010 and 2024 were included, potentially omitting relevant research in other languages or earlier periods. Third, while the proposed boiling water model and its philosophical extension offer theoretical insights, these frameworks have not yet been empirically tested. Future empirical studies are needed to validate the model's applicability across diverse clinical and cultural contexts. Nonetheless, these limitations do not diminish the conceptual contributions of this review in advancing a theory‐informed understanding of patient leaflet design.

### 4.6 Implications and Future Research Directions

The findings of this review suggest that health education leaflets should evolve from static information sheets into dynamic tools that scaffold patients' meaning‐making processes. Future work should prioritise evaluating how patients read, interpret and emotionally respond to these materials, as well as how design interventions affect decision quality, adherence and self‐management [5, 6, 7, 8]. Establishing such evidence will be key to ensuring that health education leaflets contribute not just to knowledge acquisition, but to informed, empowered and health‐literate patient behaviours.

## 5 Conclusions

In conclusion, this thematic narrative review highlights that PILs are most effective when designed to address all three levels of health literacy—functional, interactive and critical—while integrating cognitive learning principles such as Cognitive Load Theory, Multimedia Learning Theory and Dual Coding Theory. The proposed boiling water model conceptualises the reflective and decision‐making processes patients undergo, emphasising the need for sustained cognitive support to reach a ‘boiling point’ of understanding.

Adopting a user‐centred design approach, embedding evaluation mechanisms and fostering interdisciplinary collaboration can enhance the clarity, relevance and accessibility of health materials. These strategies may transform leaflets from static information carriers into dynamic tools that support informed, value‐aligned health actions and promote patient empowerment across diverse clinical contexts. Incorporating cultural sensitivity, iterative usability testing and feedback loops from both patients and healthcare providers can ensure these materials remain responsive to evolving needs. This can bridge communication gaps, reduce inequities and strengthen shared decision‐making in routine practice.

## Author Contributions

**Yung‐Hui Tang:** conceptualisation, methodology, software, validation, formal analysis, investigation, resources, data curation, writing – original draft. **Chin Tsai Lin:** validation, writing – review and editing, visualisation. **Li‐Chu Wu:** conceptualisation, validation, writing – review and editing, supervision, project administration.

## Ethics Statement

As this article is a narrative literature review that does not include human participants or individual‐level data, formal ethical approval was not required.

## Consent

The authors have nothing to report.

## Conflicts of Interest

The authors declare no conflicts of interest.

## Data Availability

All data supporting the findings of this study are included within the article and its Supporting Information.

## Summary Table of Included Articles

This appendix summarises the 28 articles included in the thematic synthesis. Each entry provides the first author and publication year, abbreviated title, source journal, study type, and the primary focus or theme addressed in the study.

**Table:**

No.	First author (Year)	Title (Shortened)	Source	Type	Main focus/Theme
1	Giannopoulos et al. [24]	Health literacy & radiation therapy	J Cancer Educ	Quantitative	Health literacy assessment
2	Ahn et al. [25]	Readability in stroke education	Top Stroke Rehabil	Review	Readability review
3	Williams et al. [26]	Ophthalmology patient materials	BMC Ophthalmol	Review	Patient comprehension
4	Haller et al. [27]	Urogynecology readability	Female Pelvic Med Reconstr Surg	Quantitative	Readability versus patient level
5	Singh et al. [5]	Innovation in readability metrics	Clin Pract	Perspective	New metrics in education
6	La Scala et al. [28]	Cochlear implant brochures	Am J Audiol	Quantitative	Parental understanding
7	Boles et al. [29]	Dental education brochures	J Dent Hyg	Quantitative	Text readability
8	Okatch et al. [30]	Lead poisoning info materials	BMC Public Health	Mixed Methods	Content clarity and risk info
9	Brütting et al. [31]	Melanoma booklet quality	J Cancer Educ	Mixed Methods	Design quality and readability
10	Chhabra et al. [32]	HPV counselling materials	Acad Pediatr	Evaluation	Health literacy perspective
11	Nicolas‐Joseph et al. [33]	Cancer immunotherapy materials	Patient Educ Couns	Evaluation	Online content quality
12	Robb et al. [34]	Spanish pelvic floor handouts	Female Pelvic Med Reconstr Surg	Quantitative	Language accessibility/readability
13	Restrepo et al. [35]	Breast cancer education online	J Cancer Surviv	Evaluation	Readability for survivors
14	Pakhchanian et al. [36]	Ophthalmic brochure readability	Semin Ophthalmol	Quantitative	Specialty‐specific readability
15	Ugas et al. [37]	Machine translation for PEM	Patient Educ Couns	Feasibility Study	Translation accuracy & accessibility
16	Duran et al. [38]	AI versus human brochures (Spanish)	Pediatr Dermatol	Comparative Analysis	AI versus human language clarity
17	Postacı and Dal [39]	LLMs for retinopathy materials	Turk J Ophthalmol	AI Tool Assessment	AI model effectiveness
18	Moore and Millar [40]	Antibiotics patient info (TB)	Lung	Evaluation	Instructional clarity in drug info
19	Breneman et al. [41]	AI enhancing readability	Arch Dermatol Res	AI Commentary	LLM role in simplifying content
20	Wright [42]	Kidney transplant listing & literacy	Nephrol Nurs J	Perspective	Health literacy in transplant settings
21	Stafford et al. [43]	Postpartum literacy outcomes	Matern Child Health J	Quantitative	Patient outcomes and literacy
22	Fortuna [44]	AMD and literacy link	OJOT	Correlational Study	Literacy and vision in elderly
23	Quintero et al. [45]	IDEA safeguards (Spanish)	Lang Speech Hear Serv Sch	Evaluation	Legal doc accessibility (Spanish)
24	Fortuna et al. [46]	Modified materials for AMD	OJOT	Intervention Study	Simplified content for AMD
25	Papadakos et al. [47]	Systemic therapy materials (Cancer)	Support Care Cancer	Multi‐Centre Survey	Evaluation of PEM quality
26	Hanspal and Hanspal [48]	Physician roles in infodemic	Fam Doctor NY State	Commentary	Provider communication in crises
27	Azios et al. [49]	Aphasia info quality online	Int J Speech Lang Pathol	Content Analysis	Digital health info quality
28	Kennard [50]	Health literacy in nursing Ed	Nurs Educ Perspect	Educational Perspective	Nursing curriculum integration
